# The co-creation of eating and wellbeing guidelines with rangatahi (young people) in Aotearoa New Zealand

**DOI:** 10.1017/S1368980025101122

**Published:** 2026-03-26

**Authors:** Renee Railton, Rachael Glassey, Eloise Goddard, David C. Tipene-Leach, Raun Makirere-Haerewa, Layla Christison, Boyd Swinburn

**Affiliations:** 1 Te Kura i Awarua Rangahau Māori Research Centre, Eastern Institute of Technology, Hawke’s Bay, New Zealand; 2 School of Population Health, University of Auckland, New Zealand

**Keywords:** Eating guidelines, Young people, Health promotion, Participatory research, Indigenous knowledge

## Abstract

**Objective:**

To co-create with rangatahi (young people) evidence-based eating and wellbeing guidelines for young people in Aotearoa New Zealand (NZ), informed by mātauranga Māori (traditional Māori knowledge).

**Design:**

Rangatahi collaborated with Māori and non-Māori experts to review existing health guidelines covering sustainable eating, physical activity, screen time, sleep and mental wellbeing and develop their own set of guidelines. Peer feedback on the draft guidelines was used to produce the final guidelines. The process integrated scientific evidence with mātauranga Māori, following tikanga Māori (Māori custom) to ensure a culturally centred process.

**Setting:**

Wānanga (learning workshops) were held at a local marae (traditional meeting house), and feedback presentations were held in four secondary schools in Hawke’s Bay, NZ.

**Participants:**

Seventeen rangatahi from four schools with high Māori student enrolment participated in the wānanga, and ninety-four students provided peer feedback through surveys.

**Results:**

The rangatahi created ten eating and ten wellbeing guideline messages. These messages were invitational (beginning ‘Let’s try to…’) acknowledging the challenging journey for many rangatahi from current to recommended behaviours. Only one quantification (8–10 h of sleep) was included. Three eating and three physical activity guidelines incorporated the concepts of ‘mauri’ (life force). The guidelines addressed contemporary issues including sustainable eating, ultra-processed foods, social dimensions of eating and physical activity, screen time and cyberbullying. They also emphasised respect, rights and responsibilities, concluding with a motivational whakatauki (proverb) about aspirations.

**Conclusions:**

Innovative, relevant and contemporary eating and wellbeing guidelines have been successfully co-created by rangatahi Māori for all young people across NZ.

The years of adolescence and youth (10–24 years) represent a unique time of life. It is a time where autonomy regarding food choice, enabled by increased purchasing power and the development of identity, becomes an increasingly salient informer of health behaviours^(1)^ and dietary habits and food preferences are developed^(2)^. As such, establishing and maintaining good nutrition and a strong sense of wellbeing are imperative as rangatahi (young people) are navigating the complexities of modern society and creating habits that will follow them into adulthood, influencing future outcomes such as physical and mental health^(3)^. The provision of effective, relevant and accessible information is, therefore, essential to helping guide healthy food choices for rangatahi.

Low food literacy, financial hardship and obesogenic environments contribute to the unhealthy eating patterns and lifestyle behaviours of rangatahi across Aotearoa New Zealand (NZ)^(4,5)^. Obesity rates in children have increased over the past 5 years with 12·5 % of children aged 2–14 years classified as obese in 2023/2024 and the prevalence of obesity in Māori children higher at 15·3 %^(6)^. It is widely recognised that NZ’s health system requires significant rebuilding to address the underlying social, economic and commercial drivers of obesity. Rangatahi perspectives on hauora (wellbeing) during the COVID-19 lockdowns of 2020/2021 have highlighted obesity and mental health as significant problems for young people^(7)^. For young New Zealanders, mental health needs have increased sharply over the last decade^(8,9)^, and 23 % of young people reported experiencing ‘high’ or ‘very high’ levels of psychological distress in 2023^(6)^. These factors may contribute to sustained nutritional and wellbeing issues for rangatahi, such as low fruit and vegetable intake, high intake of ultra-processed foods, poor oral health, high rates of obesity and declining mental health^(10)^. This highlights the need for high-impact messaging for rangatahi.

The NZ government publishes a number of food and nutrition guidelines, each developed to fit a sociocultural context and designed for New Zealanders from infancy to the elderly. The purpose of guidelines is to provide consistent, evidence-based, contemporary information for the public and health professionals on quality nutrition and eating recommendations^(11)^. However, there are important gaps within NZ guidelines. They emphasise individual responsibility and overlook social, spiritual, cultural and environmental factors that influence food choices and health behaviours, and they have no link to the sustainability of diets. Further, they do not include any aspects of mātauranga Māori (traditional Māori knowledge) and, consequently, are unlikely to meet the needs of Māori^(12)^. In addition, the current NZ healthy eating guidelines for young people were developed without input from the target population, making it unlikely that rangatahi are aware or engaged with them.

Children (ages 9 and 13 years) in the Hawke’s Bay region of NZ exhibit poor dietary habits despite the region’s reputation for its fruit and vegetable production. A recent regional study showed that very few young people meet the recommended daily intake for vegetables (13 %) or fruit (39 %), and a high proportion consume unhealthy foods daily (72–80 %)^(13)^. Hawke’s Bay has been highlighted as one of two regions in NZ that did not experience a recent decline in preschool obesity rates, with more than 33 % of all children in Hawke’s Bay being classified as overweight or obese^(14)^. In Hawke’s Bay, 37 % of school-aged children identify as Māori^(15)^, and, therefore, this region is uniquely placed to benefit from an intervention addressing eating and wellbeing in rangatahi.

Broad domains of adolescent wellbeing have been characterised and include health and nutrition, connectedness and contribution, safety and supportive environment, learning and competence/skills, and agency and resilience^(16)^. However, we could find no country with comprehensive wellbeing guidelines for young people. NZ does have has some simple ‘sit less, move more, sleep well’ messages incorporated into its heathy eating guidelines for young people. These recommend less than 2 h a day of (non-educational) screen time, an hour of moderate to vigorous activity a day and 8–10 h of sleep per night^(11)^.

Thus, evidence-based guidelines need to be co-created with rangatahi to increase engagement and uptake of healthier eating and wellbeing behaviours. A collaborative approach to developing guidelines that resonate with rangatahi could promote healthier eating, exercise, sleep and screen time habits, as well as enhance mental health and connections with Māori kai (food) and various aspects of wellbeing for rangatahi in NZ. The aim of this paper is to describe the process and outcomes of co-creating with rangatahi Māori a set of evidence-based eating and wellbeing guidelines for all young people, but particularly rangatahi Māori, which are informed by mātauranga Māori and tikanga Māori.

## Method

### Nourishing Hawke’s Bay: He wairua tō te kai

This project was a part of a wider research initiative in the Hawke’s Bay region. Launched in January 2020, the *Nourishing Hawke’s Bay (NHB): He wairua tō te kai (there is life, wellbeing and meaning to be found in food)* research project aimed to unite the Hawke’s Bay community in a shared goal of enhancing food security, with a particular focus on improving nutrition for children and young people. NHB employed mātauranga Māori and systems dynamics as lenses through which to develop the model.

### Sampling frame

Fifteen Hawke’s Bay secondary schools with the highest percentage of Māori students on their school roll were approached to participate in the programme. Schools with specific student populations such as teen parent units, correspondence schools, special schools and activity centres were excluded.

### Recruitment

School principals from the selected schools were invited to nominate Year 12 (16- to 17-year-old) rangatahi from their school who they considered would be interested in the programme. Four schools agreed to participate. The school provided the study coordinator with contact details of interested rangatahi and their parents/caregivers to provide further information, gauge interest and enrol them into the programme.

An information evening was held for whakawhanaungatanga (the making of relationships) and to outline the programme further for rangatahi and their parents/caregivers. Both parent/caregiver and rangatahi consent were required. Across all schools, a total of seventeen rangatahi consented to participate (6 male and 11 females; 16 Māori and 1 non-Māori).

### Roles of rangatahi and co-design approach

Co-design approaches which incorporate kaupapa Māori (research/services ‘by Māori for Māori’) principles are based on best practice in co-design methodologies: cultural-centredness, community engagement, systems thinking and integrated knowledge translation^(17)^. Throughout the Nourishing Hawkes Bay initiative, we wove together the knowledge bases of mātauranga Māori (traditional Māori knowledge) and systems thinking^(18,19)^. While systems thinking did not feature strongly in this project, the mātauranga Māori aspects did (described below). While the rangatahi were research participants, consenting to be involved in this project, their roles were as research translators and message co-creators. They took the research presented to them on national and international guidelines and from oral presentations and discussions and translated it into an integrated package of messages for rangatahi.

### Procedure

The programme consisted of two wānanga (learning workshops) based at a local marae (traditional meeting house) as well as peer-to-peer presentations and feedback sessions at schools. Parents and rangatahi were invited to join a private Facebook group where updates on the rangatahi activities and learnings during the programme were uploaded to allow whānau (family) engagement.

Wānanga One involved a 5-d live-in workshop held at a local marae during the July 2023 NZ school holiday break. All rangatahi were comfortable with the marae setting and its protocols, simultaneously providing an appropriate setting for the incorporation of mātauranga Māori (and tikanga Māori) into the process. Throughout the week, rangatahi worked together with health and wellbeing experts where they were introduced to the current NZ Ministry of Health and international nutrition guidelines, were tutored on how guidelines are created and participated in workshops regarding the nutrition and wellbeing practices rangatahi should be striving for. Speakers were chosen by the research team on the basis of their background, experience and expertise in the content areas and mātauranga Māori. Using the knowledge they gained throughout the week, the rangatahi co-created a draft set of eating and wellbeing guidelines consisting of twenty separate messages.

The process of developing these eating and wellbeing messages (encompassing emotional and physical safety, sleep hygiene, physical activity recommendations and screen time) is described in Table 1.

**Table 1.** Overview of wānanga One programme

Day	Activities	Presentation details
1	Whakawhanaungatanga (establishing relationships); local history; mātauranga Māori (Māori knowledge); teamwork	**Kaumātua (elder):** Local history including kai (food) stories from the past. **Māori public health physician:** history of the diet of Māori (pre-colonisation, colonisation, modern) and its health consequences; pūrākau (creation story) and mātauranga Māori concepts and ways of knowing.
2	Guideline development; basics of nutrition; international and national nutrition guidelines; mental resilience exercises; feedback activities on existing national and international guidelines; constructing and reviewing the foundations of rangatahi-developed nutrition guidelines; physical activity exercises; helping to prepare their dinner at a local Māori-owned, organic café celebrating Matariki (Māori New Year).	**Public health physician with food and nutrition expertise:** The process of developing guidelines, the research behind the recommendations and message translation, background to the international guidelines being examined. **Public health dietitian:** NZ healthy eating guidelines for young people – nutrition concepts, evidence, structure of messaging, uses of guidelines. **Dietitian trainee:** hands-on activity classifying foods, NOVA classification system and ultra-processed foods definitions and health effects.
3	Development of wellbeing guidelines; international and national guidelines on sleep, screen time and physical activity; Discussions on tūrangawaewae (a traditional place to stand); mindfulness exercises: physical activity exercises; constructing and reviewing the foundations of rangatahi-developed wellbeing guidelines.	**Renown Māori leader in physical activity and wellbeing:** discussions and exercises on respect, personal safety and tūrangawaewae. **Regional Sports Trust health promoter:** national and international (Australia, England, the USA and Canada) guidelines on screen time, sleep and physical activity. Exercises on mindfulness. **Māori sportsman and physical activity promotion practitioner:** brainstorming exercise on ‘sport’, ‘physical activity’ and ‘physical activity in the environment’.
4	Refining the wording of each of the eating and wellbeing messages; incorporating Māori health concepts; practising presentation skills; brainstorming dissemination options.	**Public health physician:** drafts of each of the eating and wellbeing guidelines. **Māori public health physician:** Māori models of health including Te Whare Tapa Wha and Te Pae Ora.
5	Pre-dawn viewing of the rise of the Matariki constellation (Pleiades) and stories of its significance for Māori; final refinement of the messages, including the whakatauaki (proverb) capstone for the guidelines; presentation of guidelines to parents.	**Kaumātua:** Matariki stories. **Māori public health physician:** background to the whakatauki capstone.

Small group activities were used to critique existing guidelines and speaker presentations, and their comments were recorded on butcher’s paper. In addition, one of the co-authors (EG) kept notes of verbal feedback and group discussions. Quotes were extracted from the written and verbal feedback from rangatahi and are presented to give a sense of the evolution in thinking from the rangatahi group as they created their own guidelines.

The wānanga was interspersed with physical activities, team-building exercises, games and other activities throughout the week to engage the rangatahi. These included a 10 km run, team bike challenge and dinner at a local restaurant where the rangatahi had access to the kitchen to learn about and create Māori kai.

During terms 3 and 4 of the NZ 2023 school year, the rangatahi tested the eating and wellbeing guidelines with other rangatahi. As a pragmatic approach to getting feedback, four schools agreed to having some of the rangatahi who co-created the guidelines present the messages to their peers in year levels 10–12. The peer groups were asked to rate each of these messages on a five-point Likert scale from ‘I strongly agree with this’ to ‘I strongly disagree with this’ straight after the presentation of each guideline message using Alchemer survey software loaded onto tablets. They were also encouraged to provide written feedback on why they chose that rating for each message.

During December 2023, after the end of the school year, the original cohort of rangatahi returned for a 3-d follow-up wānanga (Wānanga Two) held at the same marae. Similar to Wānanga One, Wānanga Two consisted of team building and physical activities and a visit to a local community garden that focused on the importance of local and sustainable food production. During the wānanga, the rangatahi were presented with an overview of the peer feedback from the school presentations. This included histograms of response frequencies across the Likert scale for each message. The open-ended comments were also summarised and presented. The rangatahi used this feedback to further refine their initial twenty guideline messages.

### Mātauranga Māori and guideline development

The lack of any appropriate cultural alignment in the current guidelines highlighted the need for better representation of Māori in guideline development. By nature of the co-design research approach, the project exercised a deliberate thoroughness which Pohatu^(20)^ poses as a mātauranga Māori relational concept, the principle of growth in relationships. The wānanga involved strong aspects of whakawhanaungatanga, tikanga Māori and pūrākau (creation stories). The use of research methods informed by Indigenous worldviews that acknowledge the reality of multiple perspectives in Te Ao Māori (Māori world view) presents an opportunity to mitigate the transcultural gap between dominant Pākehā (NZ European) worldviews and Te Ao Māori^(21)^. Oversight of this unifying process is important to ensure that mātauranga Māori is not subsumed into Eurocentric knowledge paradigms and subsequently relegated to secondary importance^(22)^. The position of cultural oversight in the co-development of guidelines with rangatahi was filled by one of the co-authors (DTL) who is a public health physician and expert cultural advisor. The information provided by the knowledge bases of mātauranga Māori is essential to the development of culturally responsive eating and wellbeing guidelines, particularly as a potential influencer of equity in health and education outcomes.

The knowledge base of mātauranga Māori additionally provided some of the key conceptual framework domains and shaped the evolution of the rangatahi guidelines throughout both wānanga and the peer feedback process. Wānanga One included presentations and workshops on knowledge extrapolated from mātauranga Māori, including topics such as food perspectives, and Durie’s^(23)^ seminal Te Whare Tapa Whā health model. These concepts informed the purpose and expression of the rangatahi guidelines, especially concerning their orientation around the concept of mauri (life force) as a measure of overall wellbeing. Local pūrākau were used to develop discussion with the rangatahi about mauri as the life force that exists within all living things and the interconnectedness of mauri with that of the whenua (land) and broader taiao (environment). Wānanga Two introduced Indigenous knowledge practices such as maramataka (Māori lunar calendar) through to learning from kaumātua (elders) in marae community gardens.

## Results

The development of the guidelines consisted of three parts: drafting of messages, peer feedback and message refinement, and naming and framing of the guidelines.

### Part 1) Drafting of messages

Wānanga One saw the rangatahi develop twenty eating and wellbeing messages that they felt were relevant to them and their peers. Ten messages were related to eating and nutrition, and ten messages were related to wellbeing.

#### Eating and nutrition

A public health dietitian presented the current NZ nutrition and wellbeing guidelines specifically available for young people. The rangatahi were asked to critique the guidelines. Excerpts from their critique are included in Box 1.

Box 1. Rangatahi critique of New Zealand’s current ‘Healthy Eating for Young People’ pamphlet

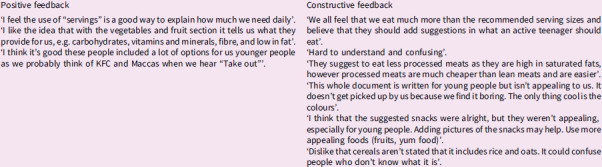

In the next session, a research facilitator led a hands-on activity focused on food processing and its impact on the nutrient profile of food products. The session covered the NOVA classification scale of foods, which considers the level of processing undergone to produce foods. The NOVA classification system consists of four groups^(24)^.

- Group 1 – unprocessed or minimally processed foods like fruit, vegetables, eggs, meat and milk;
- Group 2 – processed culinary ingredients like vegetable oils, butter, honey and sugar;
- Group 3 – processed foods, for example, canned vegetables or legumes, salted nuts and seeds, and smoked meats and fish; and
- Group 4 – ultra-processed foods, such as ice cream, chocolate, biscuits, cakes, chips and energy bars.

While many dietary guidelines have different terminologies for the types of unhealthy foods to avoid (e.g. non-core, discretionary, highly processed, high in fat, salt and sugar), several of the more recent guidelines, especially from Latin America, used the term ‘ultra-processed foods’ derived from the NOVA classification. These guidelines also had the strengths of being more holistic by including the environmental sustainability and family/social aspects of food preparation and eating which were appealing to rangatahi.

Since the NOVA classification and concept of ultra-processed foods were new to the rangatahi, a selection of foods was presented to them, which they were then required to organise into the respective NOVA food processing classification groups. The exercise stimulated discussion and understanding of how food processing can impair the product’s nutrient profile and reinforced the value of eating patterns involving mainly non- and minimally processed whole foods.

Following this, examples of food-based dietary guidelines (FBDG) from selected other countries (Brazil, Chile, Mexico, Norway and the USA) were presented to the group to understand how they construct and communicate their guidelines. The research team had previously reviewed the healthy eating guidelines from many countries^(25)^ and elected to present those from five countries (in addition to NZ) so as not to overwhelm the rangatahi. Countries were selected for different reasons. Latin American countries such as Brazil, Chile and Mexico have been at the leading edge of developing innovative guidelines that consider broader aspects associated with food consumption (e.g. sustainability, cultural and social aspects, and inclusion of ultra-processed foods). Norway was chosen for similar reasons, and the USA was selected due to their extensive evidence review processes for FBDG revision every 5 years. It should be noted that the selected countries did not have specific healthy eating guidelines dedicated to young people.

Rangatahi analysed these international examples of FBDG, identifying aspects they liked and disliked. Excerpts from their critiques are presented in Box 2.

Box 2. Rangatahi critique of some of the food-based dietary guidelines

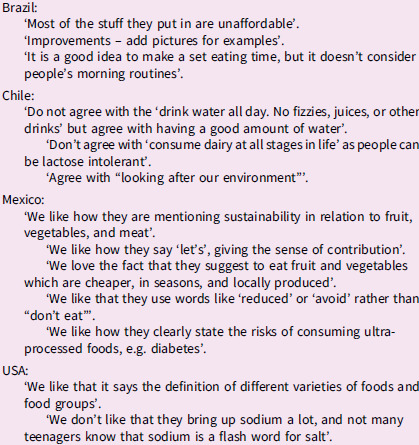

Many rangatahi liked the messaging used in Mexico’s FBDG, which featured a main sentence briefly describing the recommendation followed by a small supporting paragraph detailing the benefits and scientific background of the corresponding recommendation. Further, they liked the phrasing used in the main sentence, featuring the word ‘Let’s’ when they proposed a concept. The rangatahi felt that it was an all-encompassing, inviting approach that felt inclusive and would encourage more rangatahi to engage with the material.

Developing the foundations of the guidelines involved understanding the fundamental themes across most guidelines, for example, eating plenty of vegetables and fruits every day or emphasising whole-grain cereals over refined alternatives. The research facilitators constructed several draft messages that described these key concepts, which they then proposed to the rangatahi. Phrases were extracted from the international case studies, such as ‘Consume lots of vegetables…’ or ‘Let’s eat lots of vegetables…’ or ‘Try eating lots of vegetables…’. The rangatahi were required to vote for their preferred option, and only themes with the most votes were included in the final eating guidelines.

#### Wellbeing

While wellbeing ‘encompasses quality of life and the ability of people and societies to contribute to the world with a sense of meaning and purpose’^(26)^, for the purposes of these guidelines, we operationalised them as messages on physical activity, sleep, screentime, emotional and physical safety and aspirations.

Physical activity was an important focus, and another guest speaker facilitated an activity involving theoretical and physical parts. The rangatahi were asked to brainstorm aspects associated with four terms – ‘sport’, ‘physical activity’, ‘why we should engage with physical activity’ and ‘why not’. They were asked to consider what, why and how they associated with these terms, prompting a holistic approach to understanding why someone may or may not choose to be physically active. The rangatahi were then asked to highlight their three favourite ideas and report them to the group. Their ideas are presented in Box 3.

Box 3. Rangatahi responses to associations with sport and physical activity and why or why not they would participate.

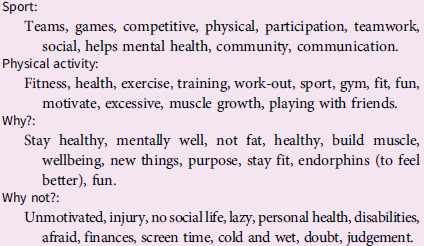

The activity was repeated, but prompt words included ‘see’, ‘know’, ‘hear’ and ‘feel’ to create a more holistic picture of being active in the environment. These ideas are summarised in Box 4.

Box 4. Holistic associations with being physically active in the environment

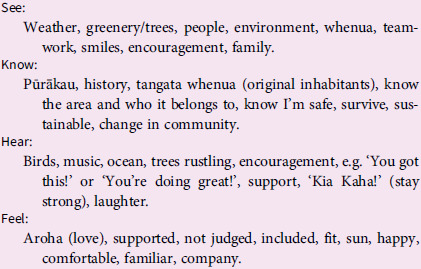

Both activities were insightful and thought-provoking for the rangatahi. It was identified that the rangatahi responses contained no prescriptive recommendations like NZ’s current physical activity recommendations (e.g. do at least an hour of moderate to vigorous activity a day). Instead, broader aspects were considered, such as being outdoors, with friends or family, or just for enjoyment. To conclude the structured brainstorming session, each group was asked to culminate their ideas into key messages detailed in Box 5.

Box 5. Rangatahi formative messages for physical activity

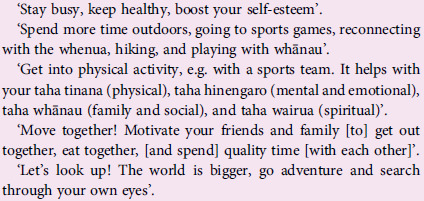

The rangatahi adopted a holistic approach when describing why they should increase their physical activity levels, considering broader aspects associated with being physically active rather than exclusively focusing on the physiological benefits. The physical activity challenges interspersed in the programme, such as a shuttle run activity to strengthen their mental capacity and an equipment carry challenge that required teamwork and communication, reminded them of the importance of mental resilience, supporting each other to complete the activities.

One guest speaker presented on screen time, sleep and physical activity recommendations nationally and internationally using Australia, England, the USA and Canada as examples. The presenter then conducted a relaxation activity with the rangatahi which encouraged mindfulness, providing positive habits for disconnecting from stressful situations to support mental wellbeing. The international examples of available wellbeing guidelines were far fewer and less well developed than the eating guidelines. They were also often in the form of evidence statements rather than having been translated into target audience messages. The rangatahi, nevertheless, felt they were important to include, and they brainstormed messages for cyber wellbeing, reducing screentime, keeping psychologically safe and showing respect.

Another presenter discussed wellbeing and the concept of tūrangawaewae (a traditional place to stand), a term that describes a place where someone feels empowered and connected, with the right to reside and belong through kinship and whakapapa (genealogy). The rangatahi were asked to consider where they felt their tūrangawaewae was, where they felt safe and had rights and responsibilities. The activity provided valuable insight into possible topics of wellbeing to include in the guidelines, identifying that rangatahi may benefit from messages that encourage reconnection with meaningful places where they feel safe and able to uphold their hauora.

As a result of these presentations and activities, the facilitators constructed a variety of wellbeing messages, including topics such as sleep, screen time, physical activity and mental and emotional health. The rangatahi again voted on the options, and the ten highest-ranked wellbeing messages were included. When deciphering how the information would be delivered in the guidelines, the rangatahi expressed that they preferred the style of Mexico’s dietary guidelines, which featured ‘Let’s’ at the beginning of each message; however, they wanted to adapt this further. They proposed starting specific messages with, ‘Let’s try to…’ as they understood that engaging with these messages might be difficult for some rangatahi, and by including ‘try to’ in the messaging, they believed they were more approachable. They also expressed that they would like to replicate Mexico’s FBDG further, by including a supporting paragraph explaining each message. The rangatahi also requested that the guidelines end with an inspirational whakatauki (proverb) which the expert cultural advisor provided.

Several of the messages, especially for wellbeing, emerged from Māori concepts and terms: mauri as a life force and energy; relationships, especially with whānau; showing reciprocity and mutual respect; tūrangawaewae as a safe place; connectedness to whenua (land) and wai (water); and ending with an aspirational whakatauki (proverb).

### Part 2) Peer feedback and message refinements

One of the outcomes from the co-design process has been the development of rangatahi leadership capacity. This was displayed in multiple ways. They were committed, thoughtful and innovative in their inputs into the development of the draft guidelines, and several of them developed sufficient public speaking confidence to present the guidelines to their peers at school, to a regional health seminar and to other groups. The purpose of these presentations was to gather structured feedback on each guideline from their peer groups.

A total of ninety-one rangatahi across four schools provided feedback on each of the messages. Of the twenty draft messages, seventeen were rated as ‘mostly agree’ or ‘strongly agree’ by over 70 % of the rangatahi and had only minor comments. For four of the eating messages, there was less agreement or there were important feedback comments indicating a lack of understanding of the message or providing suggested changes to the wording. These four messages were refined following peer feedback:

- Message 3 ‘Let’s try to eat healthy and sustainable protein foods like chicken, seafood, baked beans, and nuts instead of beef and processed food’: This concept was relatively new for rangatahi and 64 % mostly agreed or strongly agreed with the message. However, there was some resistance regarding the recommendation to ‘eat less beef’. Because beef was specifically mentioned in Message 6 detailing protein-rich foods, it was removed from Message 3. More rationale was included in the accompanying explanatory paragraph about the foods with high environmental footprints and detailed how food waste contributes to greenhouse gas footprint.
- Message 7 ‘Let’s try to avoid ultra-processed foods, like chips, sweets, and instant noodles, which are high in fat, sugar and salt’: The concept of ultra-processed foods was also relatively new for rangatahi. Those foods probably make up about half of the average diet of rangatahi, but the proportion who mostly agreed or strongly agreed with this message was 54 %. From the suggestions, this was reworded to place the rationale (high content of fat, sugar and salt) before the examples of chips, sweets and instant noodles, for ease of reading.
- Message 8 ‘Let’s try to drink lots of water throughout the day instead of fizzy drinks, juices and energy drinks’: The mostly agree or strongly agree rating for this message was 78 %. For simplicity, the phrase ‘instead of fizzy drinks, juices and energy drinks’ was shortened to ‘instead of fizzy drinks’ as that was the biggest beverage category to reduce consumption. It encompassed energy drinks and fruit juice; other high-sugar drinks (e.g. sachet powders, chocolate milk) were less commonly consumed and would have required a longer message.
- Message 9 ‘Let’s try to choose the healthier options when having takeaways or eating out and avoid fast food chains’: The mostly agree or strongly agree rating for this was 60 %. However, the original message to choose healthier takeaways was considered unrealistic given such few options in their neighbourhoods, and many rangatahi worked part-time in fast-food chain restaurants. Thus, the revised version suggests to simply have takeaways less frequently.

### Part 3) Naming and framing of the guidelines

Wānanga Two saw the rangatahi propose that the guidelines be named the Manaora Eating and Wellbeing Guidelines. Manaora is not an existing Māori word, but the rangatahi invented this neologism to bring together the concepts of mana (high standing, importance) and ora (health) to lift the value and priority of health within the minds of rangatahi as they evolve their eating and behavioural patterns through their teenage and young adult years. They also included the strapline of ‘Tihei Hauora, Tihei Mauri Ora’ as an inspirational call to behold health and wellbeing (hauora) and behold the life force of health (mauri ora). This reinforces the holistic and positive wonder or value of health, highlights the concept of mauri which appears in several messages, and is a common exclamation of celebrating the life force.

**Figure 1.**
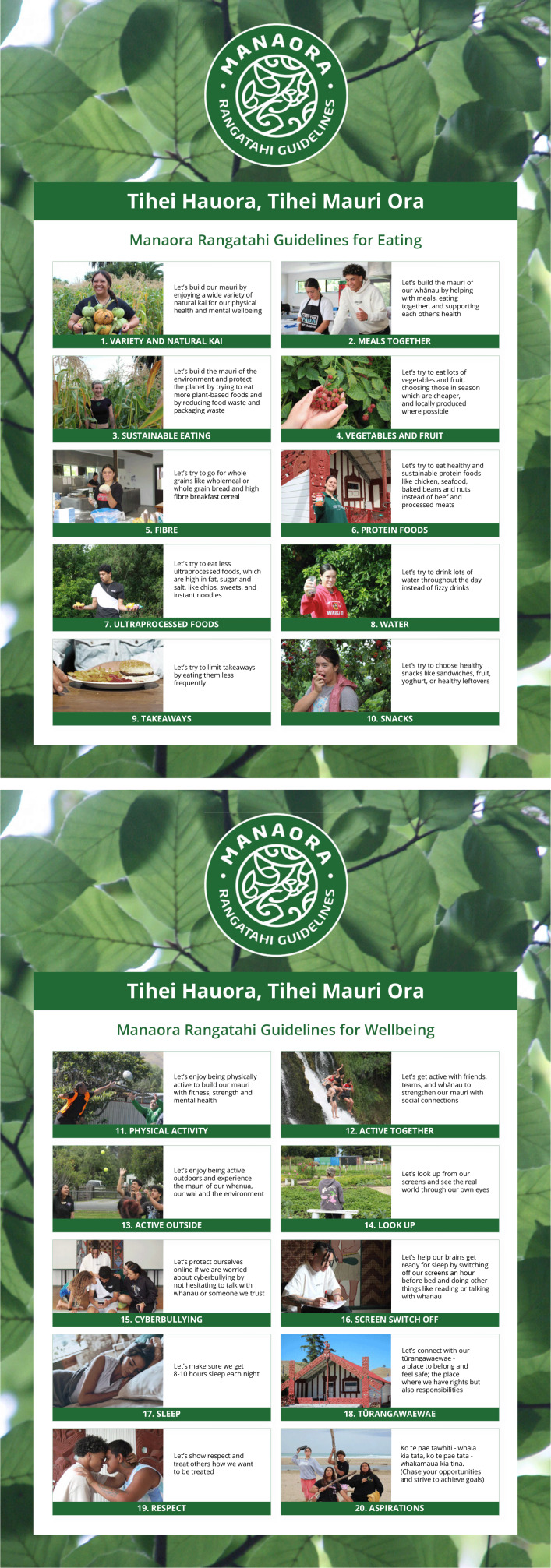
The final version of the rangatahi-developed eating and wellbeing messages ‘Manaora Rangatahi Guidelines’.

The final version of the guidelines is shown in Figure 1.

## Discussion

This project stands out for its innovative partnering with local rangatahi to co-create a set of eating and wellbeing guidelines. Unlike traditional top-down guideline development, which often results in low engagement and limited uptake among young people^(27,28)^, this project positions rangatahi as active creators rather than passive recipients of health guidance. The resulting Manaora guidelines integrate Indigenous knowledge systems with evidence-based health recommendations, utilise youth-driven messaging and framing, and incorporate current concerns such as sustainability and digital wellbeing.

The framing of the messages was invitational and aspirational (‘Let’s try to’) rather than instructive and imperative. Apart from the sleep guideline, numerical targets like serves, frequencies and durations were avoided, giving a sense of a continuum of healthy behaviours rather than specific targets. As far as possible, short rationales were included in the guideline wording, and more detailed explanations of the rationale and evidence were readily available in supplementary material. The use of Māori words and concepts throughout makes these guidelines, especially relevant for rangatahi Māori, but such terms are increasingly part of the NZ vocabulary that they will have relevance for all young people in NZ.

Co-design in research is essential, particularly in areas such as health and wellbeing where the input of the intended audience is crucial to the success of the messaging. This approach is particularly valuable when developing interventions for specific communities, as demonstrated by Thabrew *et al.* ^(29)^ in their work on eHealth interventions for young people. Similarly, Oetzel^(17)^ highlighted the effectiveness of co-design through the He Pikinga Waiora Implementation Framework, which was used to develop housing solutions that enhance health and social wellbeing for Māori. While previous studies have explored co-design with young people in health contexts such as mental health services^(30)^ and school-based health interventions^(31)^, few have integrated cultural knowledge and health recommendations. There are other models for engaging young people in research^(32–34)^, but they do not include cultural context or practical implementation strategies.

The co-design methodology used in this study differs from previous youth health initiatives. While international literature has explored health-related co-design in various areas^(28,35)^, few studies have resulted in tangible resources that bridge cultural knowledge and health science. The voice and input of the rangatahi in this project were crucial to developing effective eating and wellbeing messages that will resonate with their peers. Their active involvement in message testing and refinement of messages resulted in guidelines that speak authentically to young people as evidenced by the high acceptance of most messages during peer feedback. By empowering rangatahi to shape health and wellbeing guidelines, it increases the ownership of the content and increases the likelihood of adoption of the messaging by peers and whānau. The role of rangatahi as both designers and disseminators of the guidelines represents a novel approach to health communication recognising the avenues that young people in NZ typically use to access content, while also breaking away from traditional health education materials. This approach provides accessible, clear and relevant information about nutrition and wellbeing topics, potentially providing a model for future public health initiatives that target young people.

Particular innovations emerged in the creation of the wellbeing messages, for which there were very few international examples. These drew heavily on mātauranga Māori, and in doing so addressed most of the domains of adolescent wellbeing as defined by Ross *et al.* in their conceptual framework^(16)^. The ten messages on healthy, sustainable eating addressed the Health and Nutrition domain. The Connectedness and Contribution was also covered by several of the messages, and two focused on Safety and a Supportive Environment. The Agency and Resilience domain is threaded throughout since that is the nature of guidelines relating to individual behaviours. While the guidelines did not specifically address the Learning and Competence/Skills domain in its messages, the rangatahi participants definitely grew in their knowledge and understanding about nutrition and wellbeing.

A key strength of this work lies in the integration of mātauranga Māori with health research and the use of rangatahi as ‘translators and co-creators’ of eating and wellbeing messages for the wider NZ youth audience. The thesis is that messages designed by rangatahi Māori ensure that they are relevant to and inclusive of Māori youth but do not exclude non-Māori youth. In addition, navigating the space between published scientific research and mātauranga Māori is essential in NZ to address inequities in the health system. We have found in previous work that the incorporation of mātauranga Māori plays a pivotal role in ensuring cultural relevance, respect and community engagement^(18,19,36)^. This has certainly made the Manaora guidelines more holistic for wellbeing and more encompassing of physical, mental, social and spiritual health than any other youth-focused guidelines we found. Moreover, mātauranga Māori serves to address a number of health disparities, promoting connection to identity, and integrating cultural practices with health practices^(37–39)^. The guidelines created here show how Indigenous knowledge can enhance conventional evidence-based health recommendations. The incorporation of concepts such as mauri throughout the guidelines and the creation of the term ‘Manaora’ show how Indigenous knowledge can be applied in an innovative manner that enhances the effectiveness, relevance and acceptance of the guidelines. This approach has implications beyond NZ, offering a model for other communities seeking to develop culturally responsive approaches to health interventions.

One limitation of this work is the relatively small number of rangatahi involved in the development of the initial guidelines and the potential biases arising from this small group in one region of NZ. Larger rangatahi groups might have produced different guideline messages than those developed here, and peer testing the messages with a wider age range of young people may have provided different feedback. It will be necessary to test these messages nationally and assess awareness and uptake of the messaging after the social media dissemination campaign that was devised by the rangatahi is implemented – particularly testing the thesis that these guidelines are equally applicable to the wider audience of NZ youth. Despite these limitations, the process and resulting guidelines have highlighted the need for co-design to be vital in the development of health messages. Another limitation is that a selection of guidelines from different countries to the ones presented to the rangatahi could have resulted in different outcomes.

Our youth engagement approach was guided by tikanga Māori (Māori customs) and the considerable experience of a co-author (RMH) in running programmes and activities for young people. Sanchez *et al.* ^(40)^ identified twenty theories or frameworks for youth engagement, and mapping Indigenous approaches onto their four types of models (focus on power, process, impact or equity) would be fruitful.

The co-design approach used here offers a potential model for future health initiatives targeted at young people. This research demonstrates practical methods for integrating Indigenous knowledge with evidence-based practice to provide tangible, culturally appropriate resources for young people in NZ. Longitudinal research will be required to evaluate the long-term impact of the guidelines on health behaviours and outcomes.

Future research will explore the development of a dissemination and implementation plan for the Manaora guidelines, through social media. This will be done by supporting the rangatahi with the resources and facilities needed to develop a social marketing campaign plan and content which the rangatahi will then deliver.

This research emphasises the importance of inclusive and culturally responsive approaches to public health promotion efforts, facilitating a pathway towards more effective health initiatives in NZ. By highlighting the voices of young people, cultural knowledge and incorporating scientific evidence, the method used here not only enhances the effectiveness of health communication but also provides a model for creating evidence-based, culturally responsive health resources that engage young people. The implications extend beyond NZ, offering valuable insights for global health promotion bridging cultural knowledge and scientific evidence.

## Supporting information

10.1017/S1368980025101122.sm001 Railton et al. supplementary material Railton et al. supplementary material

## Figures and Tables

**Table:** 

Māori Term	Explanation
aroha	love
hauora	wellbeing
kaumātua	elder
kaupapa Māori	research/services by Māori for Māori
kia kaha	stay strong
marae	meeting house
maramataka	Māori lunar New Year
Matariki	Pleiades constellation (its rising marks Māori New Year)
mātauranga Māori	traditional Māori knowledge
mauri	life force
pūrākau	creation story
rangatahi	young people
taiao	environment
tangata whenua	original inhabitants
tikanga Māori	Māori customs
tūrangawaewae	a traditional place to stand
wānanga	learning workshop
whakapapa	genealogy
whānau	family
whakawhanaungatanga	the making of relationships
whakatauki	proverb
whenua	land

## Glossary

Te Reo Māori terms in the manuscript. Definitions adapted from Te Aka Online Māori Dictionary

## Supplementary material

For supplementary material accompanying this paper visit https://doi.org/10.1017/S1368980025101122
