# Supplementary material for: The co-creation of eating and wellbeing guidelines with rangatahi (young people) in Aotearoa New Zealand
Source: Public Health Nutr. 2026 Mar 26;29(1):e87. doi: 10.1017/S1368980025101122 (PMC13112303; doi:10.1017/S1368980025101122)
Supplement: Railton et al. supplementary material [file S1368980025101122sup001.pdf]

MANAORA · RANGATAHI GUIDELINES ·

Tihei Hauora, Tihei Mauri Ora

Manaora Rangatahi Guidelines for Eating Explanation

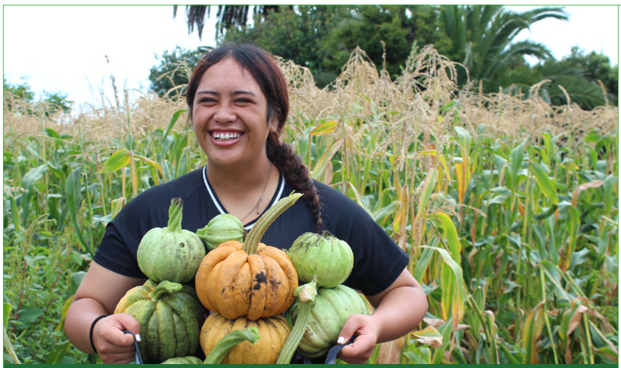

1. VARIETY AND NATURAL KAI

Let's build our mauri by enjoying a wide variety of natural kai for our physical health and mental wellbeing

EXPLANATION:

Natural kai refers to foods that are either unprocessed or minimally processed. This includes fresh, tinned, frozen and dried fruits and vegetables, as well as legumes (e.g., beans, chickpeas and peas), nuts and seeds. It is important to eat a wide range or variety of different foods to ensure that our nutritional needs are met. By consuming a variety of kai, we can protect our mental wellbeing and reduce the likelihood of developing diabetes, heart disease, cancers, and increase our life expectancy (1-3). Variety in kai also contributes to our mental wellbeing, including short-term positive effects on mood and long-term positive effects on the relationship between the gut microbiome and brain (4).

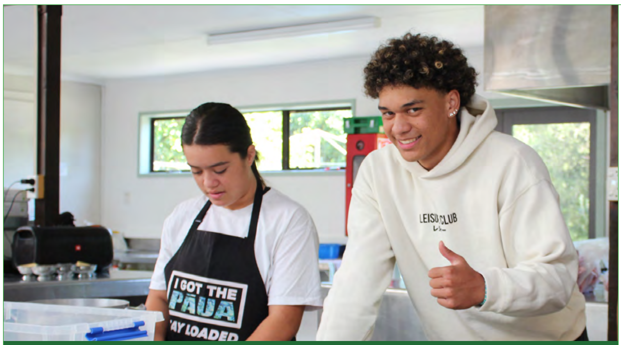

2. MEALS TOGETHER

Let's build the mauri of our whānau by helping with meals, eating together, and supporting each other's health

EXPLANATION:

The act of eating kai together, with our whānau or friends, is an important part of establishing positive rangatahi attitudes towards healthy eating through strengthening our social connections (5-6). Helping out with food preparation is another way in which we can strengthen our social connections and whanaungatanga in our whānau (7). The modelling of positive eating behaviours to whānau can also influence the quantity and quality of healthy foods eaten by rangatahi – highlighting the importance of engaging in healthy eating patterns together (8).

Tihei Hauora, Tihei Mauri Ora

Manaora Rangatahi Guidelines for Eating Explanation

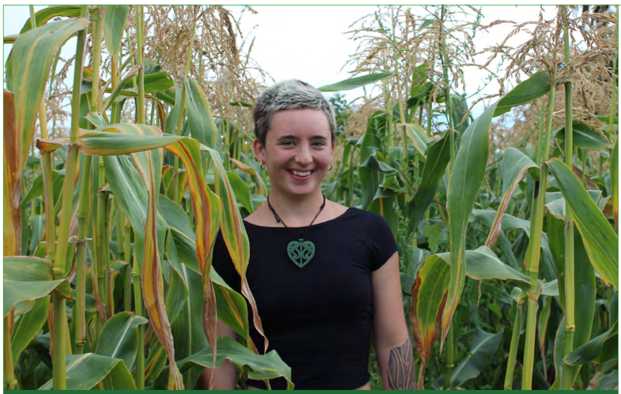

3. SUSTAINABLE EATING

Let's build the mauri of the environment and protect the planet by trying to eat more plant-based foods and by reducing food waste and packaging waste

EXPLANATION:

The foods we eat have different impacts on greenhouse gas emissions which lead to climate change (9) and can harm the health of our waterways and wildlife. The production of plant-based foods such as fruit, vegetables, legumes and nuts produce much lower emissions of greenhouse gases than foods such as processed meats, beef and dairy products (10). Increasing the number of plant-based foods in our diets can also reduce the risk of developing diabetes, heart disease and cancer (11).

In Aotearoa, food and organic waste contributes 4% of our total greenhouse gas emissions (12). Some of the emissions are preventable through increased meal planning and efficient use of food resources. Packaging waste is a major issue for the health of our taiao, particularly for our waterways (13). By avoiding buying food packaged with plastic and other non-biodegradable substitutes, we can reduce the environmental impacts of food packaging.

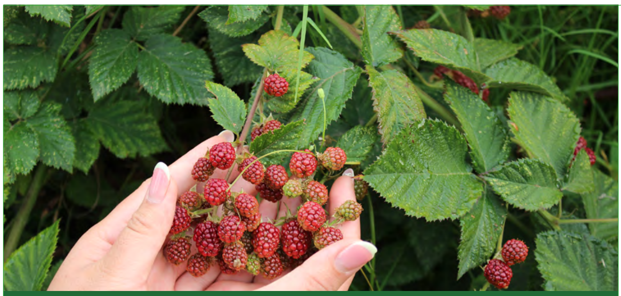

4. VEGETABLES AND FRUIT

Let's try to eat lots of vegetables and fruit, choosing those in season which are cheaper, and locally produced where possible

EXPLANATION:

Fruit and vegetables are excellent sources of many vitamins, minerals and other compounds that are necessary for good nutrition (14). Increased fruit and vegetable consumption can also promote healthy weight and mental wellbeing, and act as a protective factor against certain health conditions, including diabetes, heart disease and cancer (15). Fruit and vegetables are generally cheapest during the season in which they are harvested (e.g., apples in March) and are often tastier (16-17). Choosing produce that is locally grown can also be better for the environment, as there are reduced transport costs and associated production of greenhouse gas emissions (18).

Tihei Hauora, Tihei Mauri Ora

Manaora Rangatahi Guidelines for Eating Explanation

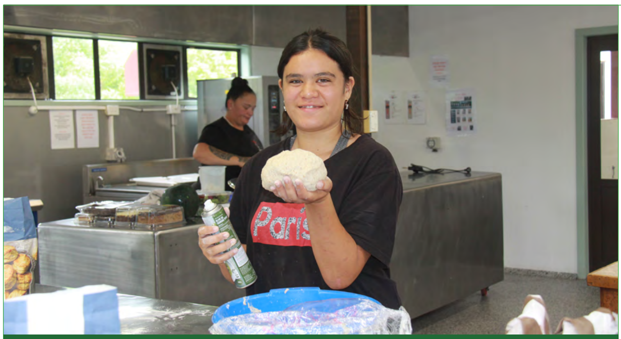

5. FIBRE

Let's try to go for whole grains like wholemeal or whole grain bread and high fibre breakfast cereal

EXPLANATION:

Fibre is a plant-based carbohydrate that cannot be completely absorbed by the gastric system. Foods that contain fibre promote healthy weight and metabolism of kai, and help protect our bodies against diabetes, cardiovascular disease and cancer (19). Increasing our fibre intake can also help with 'feeling full' and encourages more balanced serving sizes and caloric consumption. Foods such as whole grains, legumes and vegetables are common sources of fibre (20). Switching out white bread for wholegrain or wholemeal bread is one way in which we can increase our fibre consumption easily, whilst reducing the risk of type 2 diabetes (21).

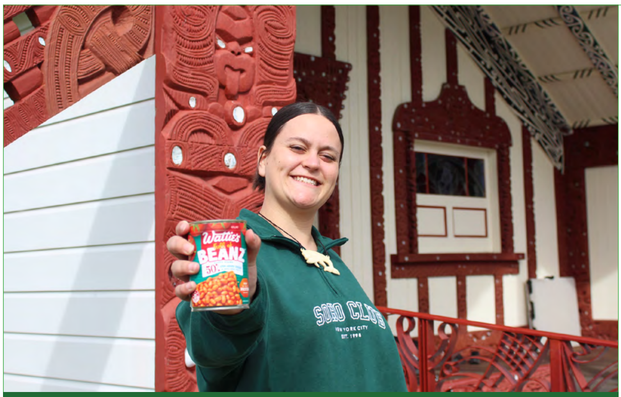

6. PROTEIN FOODS

Let's try to eat healthy and sustainable protein foods like chicken, seafood, baked beans and nuts instead of beef and processed meats

EXPLANATION:

Protein is present at varying levels in many food sources, including kaimoana, poultry, legumes and red meat. Some protein sources are healthier than others - research shows that chicken, kaimoana and plant-based protein sources are generally lower in fats and cholesterol than red meats (i.e., beef and lamb) (22). Eating a variety of both plant-based and preferred animal proteins is central to a healthy diet and can lower the risk of heart disease (23).

Protein sources also have very different environmental impacts, with ruminant animals (e.g., beef and sheep) producing the largest quantity of greenhouse gas emissions on average (24). Preferred protein sources (poultry, kaimoana and plant-based proteins) generally have lower environmental impacts and are more sustainable options with respect to greenhouse gas emissions and other environmental damage (25).

Tihei Hauora, Tihei Mauri Ora

Manaora Rangatahi Guidelines for Eating Explanation

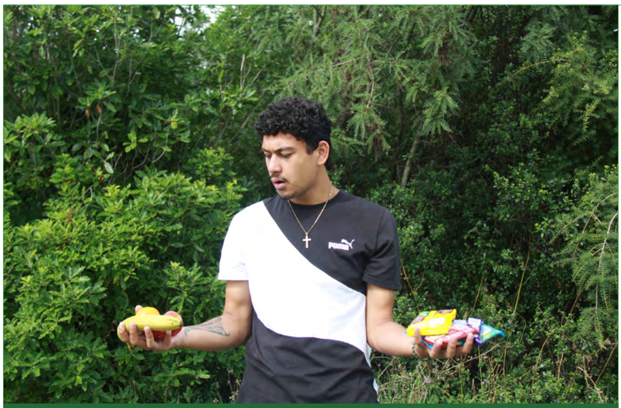

7. ULTRAPROCESSED FOODS

Let's try to eat less ultraprocessed foods, which are high in fat, sugar and salt, like chips, sweets, and instant noodles

EXPLANATION:

Ultra-processed foods are defined as food products that undergo an extensive processing procedure, often have low nutritional quality, and usually include the incorporation of artificial ingredients and additives (26). Food items categorised under the 'ultra-processed' label are also usually high in fats, sugars or salts, and are associated with an increased risk of unhealthy weight gain, poor mental health, cancers, heart disease and stroke (27-28).

As ultra-processed foods are generally 'ready-to-eat' upon purchase and are often energy-dense products, they promote overconsumption (29). These foods are also heavily packaged and produce a lot of packaging waste. The combination of overconsumption and significant packaging waste means that ultra-processed foods have a significant environmental impact and pose a risk to biodiversity globally (30-31).

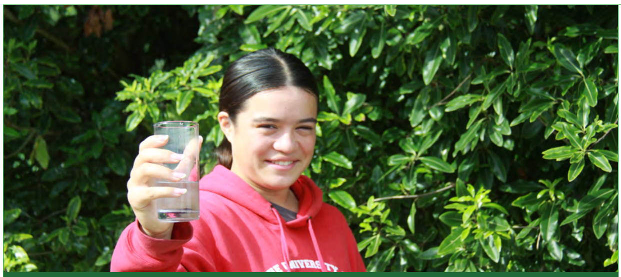

8. WATER

Let's try to drink lots of water throughout the day instead of fizzy drinks

EXPLANATION:

Fizzy drinks have a high sugar content and play a large role in unhealthy weight gain and the development of diseases such as diabetes, metabolic syndrome and tooth decay (32-33). Water is a better option for our teeth and overall health, as well as promoting energy and attention levels through necessary hydration (34). Drinking tap water from reusable containers is better for our environment than bottled water, as it reduces plastic waste.

MANAORA RANGATAHI GUIDELINES

Tihei Hauora, Tihei Mauri Ora

Manaora Rangatahi Guidelines for Eating Explanation

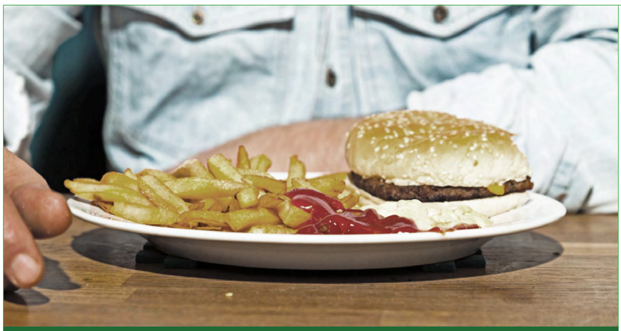

9. TAKEAWAYS

Let's try to limit takeaways by eating them less frequently

EXPLANATION:

Takeaway foods are generally high in fats, salts and sugars and can increase the likelihood of unhealthy weight gain, poor mental health and the development of illnesses such as diabetes and heart disease – as well as adversely affecting our immune systems (35). Choosing to eat takeaways also removes the capacity for our whānau to prepare food together – eliminating the opportunity to help rangatahi build healthy eating habits and whanaungatanga.

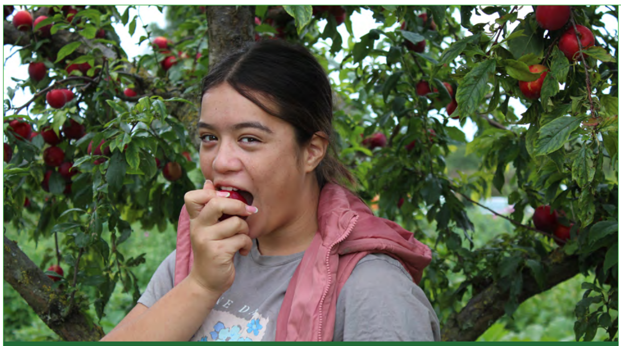

10. SNACKS

Let's try to choose healthy snacks like sandwiches, fruit, yoghurt, or healthy leftovers

EXPLANATION:

The most commonly consumed snack foods are often either ultra-processed and nutrient-poor or contain overly large quantities of fats and sugars (36). Unhealthy snacks like lollies, chips, instant noodles and sugary drinks can have negative effects on energy levels and concentration, whereas healthy snacks are a great way to maintain our energy levels throughout the day - also providing increased cognitive function and improved overall mood (37). A good way to ensure that your whānau are choosing healthy snacks and improving energy levels is to make your own at home! This could be as simple as making sandwiches, preparing fruit and vegetables, or making cheese and crackers.

Tihei Hauora, Tihei Mauri Ora

Manaora Rangatahi Guidelines for Wellbeing Explanation

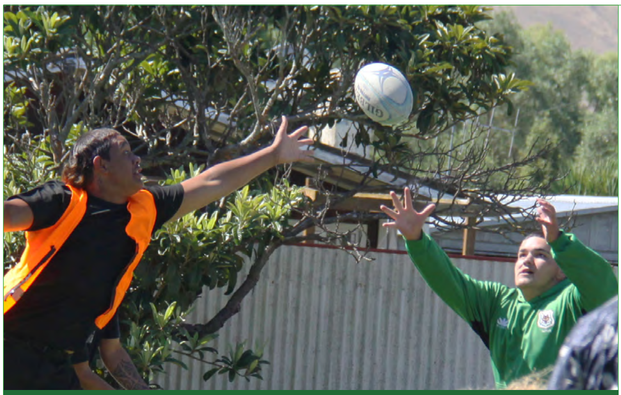

11. PHYSICAL ACTIVITY

Let's enjoy being physically active to build our mauri with fitness, strength and mental health

EXPLANATION:

Physical activity is an important part of looking after our wellbeing and comes in many shapes and forms - meaning it can be adapted to the abilities of all whānau members. Physical activity is essential to our physical wellbeing and is good for heart health, maintaining healthy weight and general fitness, as well as being a protective factor for illnesses such as diabetes, heart disease and cancer (38). There are also notable mental health benefits provided by physical activity, including reduced risk of anxiety and mood orders (39). Physical activity also acts as a mental health promoter due to the stimulation and release of natural endorphins and has a positive impact on mood and sleep quality (40).

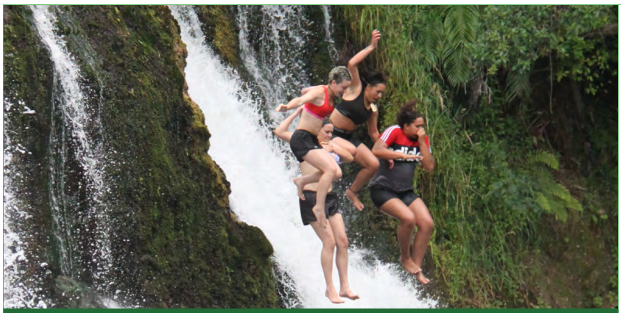

12. ACTIVE TOGETHER

Let's get active with friends, teams, and whānau to strengthen our mauri with social connections

EXPLANATION:

Social connectedness is an important part of our mental wellbeing, but also provides many benefits when paired with physical activity. Exercising with friends, whānau and teams has been shown to enhance motivation levels, self-esteem and general enjoyment of physical activity (41). Other positive impacts include feeling less fatigued after exercise and increased feelings of calmness (42). Getting active with others can also provide opportunities to meet new people and form new social connections, as well as chances to try different forms of exercise.

Tihei Hauora, Tihei Mauri Ora

Manaora Rangatahi Guidelines for Wellbeing Explanation

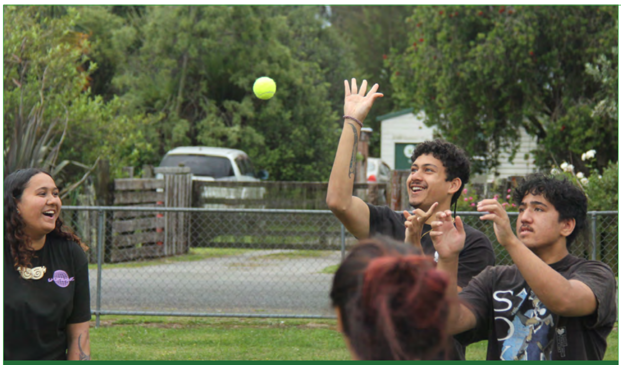

13. ACTIVE OUTSIDE

Let's enjoy being active outdoors and experience the mauri of our whenua, our wai and the environment

EXPLANATION:

There are many benefits to getting active outdoors compared with indoors, including improved mental function, decreased stress levels and positive effects on mood (43-44). Spending time in our outdoor environments with our friends and whānau also fosters stronger interpersonal relationships and connections with our environment (45). Spending time in natural bodies of water (awa, moana and roto) is also beneficial for our mental and physical health, with studies showing increased mindfulness and reduced physical stress symptoms such as lower heart rate and blood pressure (46-47).

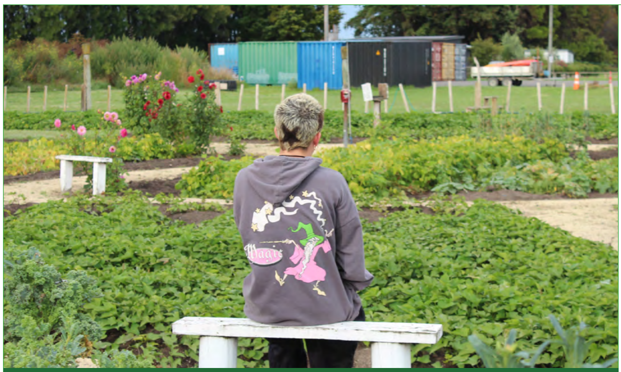

14. LOOK UP

Let's look up from our screens and see the real world through our own eyes

EXPLANATION:

Excessive screen time can harm the cognitive development of our tamariki and rangatahi, with studies indicating negative effects on the physical structure of the brain in youth (48-49). There are also physical impacts of excessive screen time, including strained eyes, reduced sleep quality and increased risk factors for heart disease and depression (50). Although devices can offer another way to strengthen our social connections, it is important that we manage our usage to look after our hauora and strengthen our real-world social connections with whānau and friends.

Tihei Hauora, Tihei Mauri Ora

Manaora Rangatahi Guidelines for Wellbeing Explanation

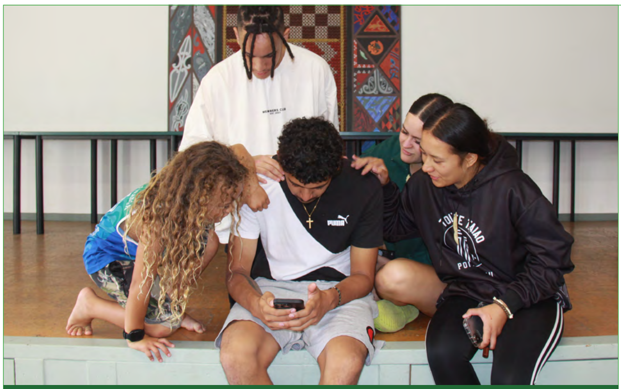

15. CYBERBULLYING

Let's protect ourselves online if we are worried about cyberbullying by not hesitating to talk with whānau or someone we trust

EXPLANATION:

As we increasingly integrate technology, the internet, and social media platforms into our daily lives, it is important to learn how to keep ourselves safe in online spaces. Cyberbullying is common in online spaces, with 46% of 18-19 year olds and 18% of all teenagers (aged 12-18) in Aotearoa reporting a cyberbullying experience (51). It is important that we talk about cyberbullying and seek help when experiencing it as there can be harmful consequences to our mental health. Studies show that there is significant link between cyberbullying and depression, anxiety and negative moods (52). Organisations like Netsafe offer free resources to learn about cyberbullying and provide some support services to individuals who have experienced it. Another option is to seek help and advice from someone you trust – this could be whānau, teachers at kura or friends.

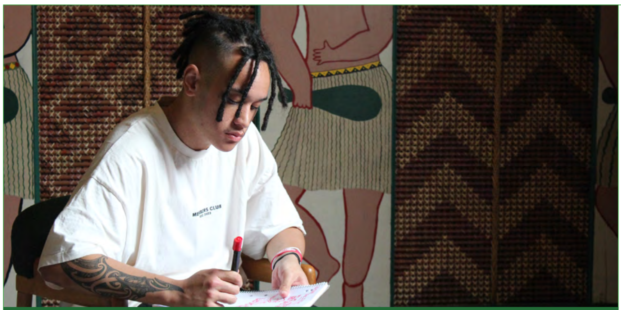

16. SCREEN SWITCH OFF

Let's help our brains get ready for sleep by switching off our screens an hour before bed and doing other things like reading or talking with whanau

EXPLANATION:

Using screens before bed can reduce the quality and length of our sleep, which subsequently increases the risk of poorer physical and mental health outcomes (53). The blue light emitted from screens has been shown to disturb our bodies' natural sleep cycles, and affects the production of our sleep hormone, melatonin (54). Instead of using screens before bed, we can try alternatives like reading pukapuka and magazines/newspapers, korero with whānau or playing board/card games.

Tihei Hauora, Tihei Mauri Ora

Manaora Rangatahi Guidelines for Wellbeing Explanation

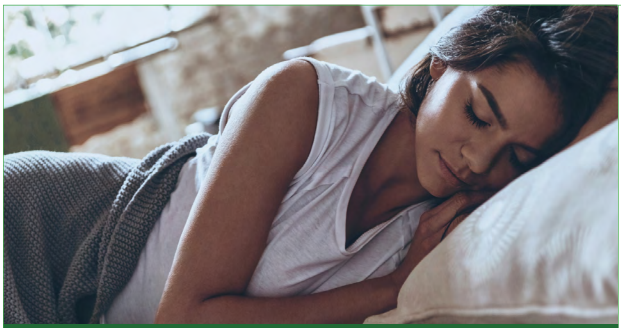

17. SLEEP

Let's make sure we get 8-10 hours sleep each night

EXPLANATION:

Good quality and duration of sleep is essential to the physical and mental development of our tamariki and rangatahi, with an abundance of research citing the importance of getting 8-10 hours of sleep each night (55). Individuals that do not get 8-10 hours of sleep each night are at increased risk of psychiatric disorders, decreased mental function, poor heart health and reduced digestive health (56). Getting enough sleep also positively affects our moods, energy levels and emotional regulation – which are all major factors in general wellbeing and quality of life (57).

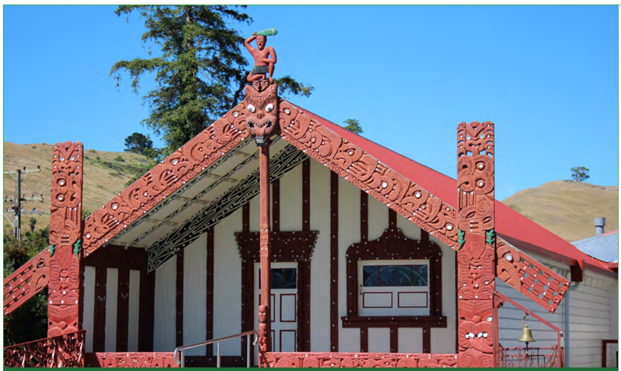

18. TŪRANGAWAEWAE

Let's connect with our tūrangawaewae - a place to belong and feel safe; the place where we have rights but also responsibilities

EXPLANATION:

Connecting with our tūrangawaewae enables self-empowerment and fosters connections with our physical and social environments, acting as a protective factor for our mental and spiritual wellbeing and overall hauora (58-59). Tūrangawaewae is sustained through whanaungatanga, relying on the connections we form with people and places to create a space to belong and feel safe (59). The places that we call tūrangawaewae differ between individuals, for some people it might be their homes, marae or other important places in their whakapapa (i.e., maunga, awa and moana) or daily life (e.g., kura or club sportsgrounds). Our tūrangawaewae is a place in which we can stand and say we belong, we feel connected, and we feel solidarity.

Tihei Hauora, Tihei Mauri Ora

Manaora Rangatahi Guidelines for Wellbeing Explanation

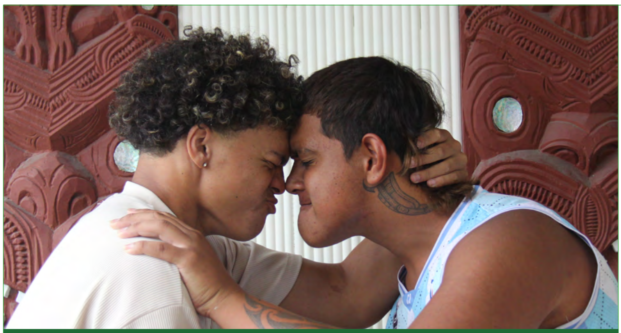

19. RESPECT

Let’s show respect and treat others how we want to be treated

EXPLANATION:

Our experiences of receiving and giving respect influence our mental and physical health, with studies showing that a lack of perceived respect increases stress levels, the risk of depression and overall physical ill-health (60). Showing respect to one another is good for our personal and communal relationships, with higher levels of respect reflecting better social engagement, self-esteem and functioning of group dynamics (60-61).

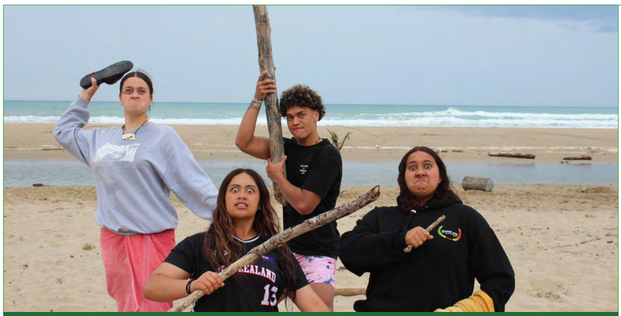

20. ASPIRATIONS

Ko te pae tawhiti - whāia kia tata, ko te pae tata - whakamaua kia tina.
(Chase your opportunities and strive to achieve goals)

EXPLANATION:

Setting goals and pursuing aspirations has a positive effect on internal motivation and is a factor in educational achievement for rangatahi (62-63). It is important that when setting goals, we attempt to make small changes to our behaviours, as it provides the best chance of establishing long-term habits (64). Simply attempting to change our behaviours has been shown to increase the likelihood of long-term behaviour change – with goal-setting an influential factor in this process (65-66).

Tihei Hauora, Tihei Mauri Ora

Bibliography

1. Kant A. Indexes of Overall Diet Quality: A Review. J American Diab Assoc [Internet]. 1996;96(8):785-791. Available from: https://www.sciencedirect.com/science/article/pii/S0002822396002179?via%3Dihub
2. López-González L, Becerra-Tomás N, Babio N, Martínez-González MA, Díaz-López A, Corella D, et al. Variety in fruits and vegetables, diet quality and lifestyle in an older adult mediterranean population. Clin Nutr [Internet]. 2021;40(4):1510-1518. Available from: https://www.sciencedirect.com/science/article/pii/S0261561421000984#sec4
3. Krebs-Smith SM, Smiciklas-Wright H, Guthrie HA, Krebs-Smith J. The effects of variety in food choices on dietary quality. J American Diab Assoc [Internet]. 1987;87(7):897-903. Available from: https://www.sciencedirect.com/science/article/abs/pii/S0002822321032120
4. Firth J, Gangwisch JE, Borsini A, Wootton RE, Mayer EA. Food and mood: how do diet and nutrition affect mental wellbeing? BMJ [Internet]. 2020;369. Available from: https://www.bmj.com/content/369/bmj.m2382
5. Neely E, Walton M, Stephens C. Young people's food practices and social relationships. A thematic synthesis. Appetite [Internet]. 2014;82:50-60. Available from: https://www.sciencedirect.com/science/article/pii/S0195666314003626?via%3Dihub
6. Walton K, Horton NJ, Rifas-Shiman SL. Exploring the Role of Family Functioning in the Association Between Frequency of Family Dinners and Dietary Intake Among Adolescents and Young Adults. Nutri, Obes and Excer [Internet]. 2018;1(7). Available from: https://jamanetwork.com/journals/jamanetworkopen/fullarticle/2715616
7. Farmer N, Cotter EW. Well-Being and Cooking Behavior: Using the Positive Emotion, Engagement, Relationships, Meaning, and Accomplishment (PERMA) Model as a Theoretical Framework. Front Psychol [Internet]. 2021;12. Available from: https://www.frontiersin.org/journals/psychology/articles/10.3389/fpsyg.2021.560578/full
8. Robinson E, Blissett J, Higgs S. Social influences on eating: implications for nutritional interventions. Nutri Research Rev. 2013;26(2):166–76. Available from: https://www.cambridge.org/core/journals/nutrition-research-reviews/article/social-influences-on-eating-implications-for-nutritional-interventions/656A232A04C591BDCD7825B133D9E6DC
9. Muscius AA, Wang DD, Janiszewski M, Eshel G, Blondin SA, Willett W, et al. Health and environmental impacts of plant-rich dietary patterns: a US prospective cohort study. The Lanc Plan Health [Internet]. 2022;6(11):892-900. Available from: https://www.sciencedirect.com/science/article/pii/S2542519622002431#cesec80
10. Clark MA, Springmann M, Hill J, Tilman D. Multiple health and environmental impacts of foods. PNAS [Internet]. 2019;116(46):23357-23362. Available from: https://www.pnas.org/doi/full/10.1073/pnas.1906908116
11. Lynch H, Johnston C, Wharton C. Plant-Based Diets: Considerations for Environmental Impact, Protein Quality, and Exercise Performance. Nutrients [Internet]. 2018;10(12). Available from: https://www.mdpi.com/2072-6643/10/12/1841
12. Ministry for Environment [Internet]. Reducing food waste; 2023. Available from: https://environment.govt.nz/what-government-is-doing/areas-of-work/waste/reducing-food-waste/
13. Kan M, Miller SA. Environmental impacts of plastic packaging of food products. Resources, Conservation and Recycling [Internet]. 2022; 180. Available from: https://www.sciencedirect.com/science/article/pii/S0921344922000040?via%3Dihub
14. Slavin JL, Lloyd B. Heath Benefits of Fruits and Vegetables. Adv Nutr [Internet]. 2012;3(4):506-516. Available from: https://www.sciencedirect.com/science/article/pii/S2161831322010262?via%3Dihub
15. Harvard [Internet]. Boston (USA): The Nurtition Source: Vegetables and Fruits; [date unknown]. Available from: https://www.hsph.harvard.edu/nutritionsource/what-should-you-eat/vegetables-and-fruits/
16. Consumer NZ [Internet]. Saving money on produce [place unknown]. Consumer New Zealand; 2018. Available from: https://www.consumer.org.nz/articles/saving-money-on-produce

Tihei Hauora, Tihei Mauri Ora

17. Miller SR, Knudson WA. Nutrition and Cost Comparisons of Select Canned, Frozen, and Fresh Fruits and Vegetables. American Journal of Lifestyle Medicine [Internet]. 2014;8(6):430-437. Available from: https://journals-sagepub-com.ezproxy.auckland.ac.nz/doi/10.1177/1559827614522942
18. Michalský M, Hooda PS. Greenhouse gas emissions of imported and locally produced fruit and vegetable commodities: A quantitative assessment. Environment Science & Policy [Internet]. 2015;48:32-43. Available from: https://www.sciencedirect.com/science/article/pii/S1462901114002469?via%3Dihub
19. Slavin J, Green H. Dietary fibre and satiety. Nutrition Bulletin [Internet];32(s1):32-34. Available from: https://onlinelibrary.wiley.com/doi/10.1111/j.1467-3010.2007.00603.x
20. Ministry of Health. Eating and Activity Guidelines for New Zealand Adults [Internet]. Wellington (NZ): Ministry of Health; 2020. Available from: https://www.health.govt.nz/system/files/documents/publications/eating-activity-guidelines-new-zealand-adults-updated-2020-oct22.pdf
21. Pepa GP, Vetrani C, Vitale M, Riccardi G. Wholegrain Intake and Risk of Type 2 Diabetes: Evidence from Epidemiological and Intervention Studies. Nutrients [Internet]. 2018;10(9). Available from: https://www.mdpi.com/2072-6643/10/9/1288
22. Zhong VW, Allen NB, Greenland P, Carnethon MR, Ning H, Wilkins JT, et al. Protein foods from animal sources, incident cardiovascular disease and all-cause mortality: a substitution analysis. Int J Epid [Internet]. 2021;50(1):223-233. Available from: https://academic.oup.com/ije/article/50/1/223/6067620?login=true
23. Harvard [Internet]. Boston (USA): The Nutrition Source: Protein; [date unknown]. Available from: https://www.hsph.harvard.edu/nutritionsource/what-should-you-eat/protein/#:~:text=However%2C%20researchers%20found%20that%20diets,to%20diets%20with%20red%20meat
24. Wanapat M, Cherdthong A, Phesatcha K, Kang S. Dietary sources and their effects on animal production and environmental sustainability. Animal Nutrition [Internet]. 2015; 1(30):96-103. Available from: https://www.sciencedirect.com/science/article/pii/S2405654515300160#sec6
25. Aiking, H. Future protein supply. Trends in Food Science and Technology [Internet]. 2011;22(2-3):112-120. Available from: https://www.sciencedirect.com/science/article/pii/S092422441000107X#sec13
26. Monteiro CA, Cannon G, Levy RB, Moubarac JC, Louzada MLC, Rauber F, et al. Ultra-processed foods: what they are and how to identify them. Public Health Nutrition [Internet]. 2019;22(5):936-941. Available from: https://www.cambridge.org/core/journals/public-health-nutrition/article/ultraprocessed-foods-what-they-are-and-how-to-identify-them/E6D744D714B1FF09D5BCA3E74D53A185
27. Ares G, Vidal L, Allegue G, Giménez A, Bandeira E, Moratorio X, et al. Consumers' conceptualization of ultra-processed foods. Appetite [Internet]. 2016;105:611-617. Available from: https://www.sciencedirect.com/science/article/pii/S0195666316302562#sec5
28. Chen X, Zhang Z, Yang H, Qiu P, Wang H, Wang F, et al. Consumption of ultra-processed foods and health outcomes: a systematic review of epidemiological studies. Nutrition Journal [Internet]. 2020;19(86). Available from: https://nutritionj.biomedcentral.com/articles/10.1186/s12937-020-00604-1#citeas
29. Mackay S, Ni Mhurchu C, Swinburn B, Eyles H, Young L, Gontijo de Castro T. State of the Food Supply [Internet]. Auckland (NZ): University of Auckland; 2019. Available from: file:///C://Users/lchristison/Downloads/State%20of%20the%20Food%20Supply%20University%20of%20Auckland%202019.pdf
30. García S, Pastor R, Monserrat-Mesquida M, Álvarez-Álvarez L, Rubín-García M, Ángel Martínez-González M, et al. Ultra-processed foods consumption as a promoting factor of greenhouse gas emissions, water, energy, and land use: A longitudinal assessment. Science of The Total Environment [Internet]. 2023;891. Available from: https://www.sciencedirect.com/science/article/pii/S0048969723030383#:~:text=Climate%20change%2C%20air%20pollution%2C%20biodiversity,et%20al.%2C%202022b
31. Leite FHM, Khandpur N, Andrade GC, Anastasiou K, Baker P, Lawrence M, et al. Ultra-processed foods should be central to global food systems dialogue and action on biodiversity. BMJ [Internet]. 2022;7(3). Available from: https://gh.bmj.com/content/7/3/e008269
32. Sundborn G, Thornley S, Merriman TR, Lang B, King C, Lanspa MA, et al. Are Liquid Sugars Different from Solid Sugar in Their Ability to Cause Metabolic Syndrome? Obesity [Internet]. 2019; 27(6):879-887. Available from: https://onlinelibrary.wiley.com/doi/10.1002/oby.22472
33. Thamassebi JF, Duggal MS, Malik-Kotru G, Curzon MEJ. Soft drinks and dental health: A review of the current literature. Journal of Dentistry [Internet]. 2006;34(1):2-11. Available from: https://www.sciencedirect.com/science/article/pii/S0300571204001885?casa_token=oWeKpsKBATAAAAAA:inwGNeGJx4W0ziSqEJttBd5stMzcaJe8j7vq459EanjAvB3M4S7-f4vM2UAYttrh2pl_LUMLtDo

Tihei Hauora, Tihei Mauri Ora

34. Fadda R, Rapinett G, Grathwohl D, Parisi M, Fanari R, Calò CM, et al. Effects of drinking supplementary water at school on cognitive performance in children. 2012;59(3):730-737. Available from: https://www.sciencedirect.com/science/article/pii/S0195666312002292?via%3Dihub
35. Myles IA. Fast food fever: reviewing the impacts of the Western diet on immunity. Nutrition Journal [Internet]. 2014;13. Available from: https://nutritionj.biomedcentral.com/articles/10.1186/1475-2891-13-61#Sec16
36. Harvard [Internet]. Boston (USA): The Nutrition Source: The Science of Snacking; 2021. Available from: https://www.hsph.harvard.edu/nutritionsource/snacking/
37. Smith AP, Rogers R. Positive Effects of a Healthy Snack (Fruit) Versus an Unhealthy Snack (Chocolate/Crisps) on Subjective Reports of Mental and Physical Health: A Preliminary Intervention Study. Frontiers in Nutrition [Internet]. 2014;1. Available from: https://www.frontiersin.org/articles/10.3389/fnut.2014.00010/full
38. World Health Organization. Physical activity [Internet]. [Place unknown]: WHO; 2022. Available from: https://www.who.int/news-room/fact-sheets/detail/physical-activity
39. Saxena S, Ommeren MV, Tang KC, Armstrong TP. Mental health benefits of physical activity. Journal of Mental Health [Internet]. 2005;14(5):445-451. Available from: https://www.tandfonline.com/doi/full/10.1080/09638230500270776
40. Fox KR. The influence of physical activity on mental well-being. Public Health Nutrition [Internet]. 1999;2(3a):411-418. Available from: https://www.cambridge.org/core/journals/public-health-nutrition/article/influence-of-physical-activity-on-mental-wellbeing/3C363AEECE5C8CAC490A585BA29E6BF8
41. Kanamori S, Takamiya T, Inoue S, Kai Y, Kawachi I, Kondo K. Exercising alone versus with others and associations with subjective health status in older Japanese: The JAGES Cohort Study. Scientific Reports [Internet]. 2016;6. Available from: https://www.nature.com/articles/srep39151
42. Plante TG, Coscarelli L, Ford M. Does Exercising with Another Enhance the Stress-Reducing Benefits of Exercise? International Journal of Stress Management [Internet]. 2001;8:201-213. Available from: https://link.springer.com/article/10.1023/A:1011339025532
43. Boere K, Lloyd K, Binsted G, Krigolson OE. Exercising is good for the brain but exercising outside is potentially better. Scientific Reports [Internet]. 2023;8. Available from: https://www.nature.com/articles/s41598-022-26093-2
44. Coon JT, Boddy K, Stein K, Whear R, Barton J, Depledge MH. Does Participating in Physical Activity in Outdoor Natural Environments Have a Greater Effect on Physical and Mental Wellbeing than Physical Activity Indoors? A Systematic Review. Environmental Science & Technology [Internet]. 2011;45(5):1761-1772. Available from: https://pubs-acs-org.ezproxy.auckland.ac.nz/doi/full/10.1021/es102947t
45. Weir K. Nurtured by nature. Monitor on Psychology [Internet]. 2020;51(3). Available from: https://www.apa.org/monitor/2020/04/nurtured-nature
46. Oliver DM, McDougall CW, Robertson T, Grant B, Hanley N, Quilliam RS. Self-reported benefits and risks of open water swimming to health, wellbeing and the environment: Cross-sectional evidence from a survey of Scottish swimmers. PLoS One [Internet]. 2023;18(8). Available from: https://journals.plos.org/plosone/article?id=10.1371/journal.pone.0290834
47. Overbury K, Conroy BW, Marks E. Swimming in nature: A scoping review of the mental health and wellbeing benefits of open water swimming. Journal of Environmental Psychology [Internet]. 2023;90. Available from: https://www.sciencedirect.com/science/article/pii/S0272494423001214
48. Gastaud LM, Trettim JP, Scholl CC, Rubin BB, Coelho FT, Krause GB. Screen time: Implications for early childhood cognitive development. Early Human Development [Internet]. 2023;183. Available from: https://www-sciencedirect-com.ezproxy.auckland.ac.nz/science/article/pii/S0378378223000889?via%3Dihub
49. Ministry of Social Development. Effects of screen time on preschool health and development [Internet]. Wellington (NZ): MSD; 2019. Available from: https://www.msd.govt.nz/documents/about-msd-and-our-work/publications-resources/research/screen-time-on-preschoolers/children-and-families-research-fund-report-effects-of-screen-time-on-p....pdf
50. Nakshine VS, Thute P, Khatib MN, Sarkar B. Increased Screen Time as a Cause of Declining Physical, Psychological Health, and Sleep Patterns: A Literary Review. Cureus [Internet]. 2022;14(10). Available from: https://www.cureus.com/articles/112862-increased-screen-time-as-a-cause-of-declining-physical-psychological-health-and-sleep-patterns-a-literary-review#!/

Tihei Hauora, Tihei Mauri Ora

51. Sense Partners. Cyberbullying in New Zealand: Estimating Social Costs. [Place unknown]: Netsafe; 2018. Available from: https://netsafe.org.nz/wp-content/uploads/2019/11/Cyberbullying-in-New-Zealand-Societal-Cost.pdf
52. Nixon CL. Current perspectives: the impact of cyberbullying on adolescent health. Adolesc Health Med Ther [Interpret]. 2014;5:143-158. Available from: https://www.ncbi.nlm.nih.gov/pmc/articles/PMC4126576/
53. Orzech KM, Grandner MA, Roane BM, Carskadon MA. Digital media use in the 2 h before bedtime is associated with sleep variables in university students. Computers in Human Behaviour [Internet]. 2016;55(part A):43-50. Available from: https://www.sciencedirect.com/science/article/pii/S0747563215301230
54. Wahl S, Engelhardt M, Schaupp P, Lappe C, Ivanon IV. The inner clock—Blue light sets the human rhythm. J Biophotonics [Internet]. 2019;12(12). Available from: https://www.ncbi.nlm.nih.gov/pmc/articles/PMC7065627/
55. Galván A. The Need for Sleep in the Adolescent Brain. Trends in Cognitive Sciences [Internet]. 2020;24(1):79-89. Available from: https://www.sciencedirect.com/science/article/pii/S1364661319302608?via%3Dihub
56. Chaput J, Gray CE, Poitras VJ, Carson V, Gruber R, Olds T, et al. Systematic review of the relationships between sleep duration and health indicators in school-aged children and youth. Applied Physiology, Nutrition, and Metabolism [Internet]. 2016;41(6):266-282. Available from: https://cdnsciencepub.com/doi/full/10.1139/apnm-2015-0627
57. Barber LK, Munz DC. Consistent-sufficient sleep predicts improvements in self-regulatory performance and psychological strain. Stress & Health [Internet]. 2010;27(4):314-324. Available from: https://onlinelibrary.wiley.com/doi/10.1002/smi.1364
58. Eckenwiler L. Displacement and solidarity: An ethic of place-making. Bioethics [Internet]. 2018;32(9):562-568. Available from: https://onlinelibrary.wiley.com/doi/10.1111/bioe.12538
59. Groot S, Hodgetts D, Nikora LM, Rua M. Tōku Tūrangawaewae: Culture, Identity, and Belonging for Māori Homeless People. In Te Rito JS, Healy SM, editors. 4th International Traditional Knowledge Conference [Internet]; 2010 June. Auckland (NZ): Knowledge Exchange Programme of Ngā Pae o te Māramatanga; 2010. 125-133. https://researchcommons.waikato.ac.nz/handle/10289/6300
60. Huo YJ, Binning KR. Why the Psychological Experience of Respect Matters in Group Life: An Integrative Account. Sociology and Personality Psychology Compass [Internet]. 2008;2(4). Available from: https://compass-onlinelibrary-wiley-com.ezproxy.auckland.ac.nz/doi/10.1111/j.1751-9004.2008.00129.x
61. Ellemers N, Sleebos E, Stam D, de Gilder D. Feeling Included and Valued: How Perceived Respect Affects Positive Team Identity and Willingness to Invest in the Team. BJM [Internet]. 2013;24(1):21-37. Available from: https://onlinelibrary-wiley-com.ezproxy.auckland.ac.nz/doi/10.1111/j.1467-8551.2011.00784.x
62. Gorard S, See BH. The Impact of Attitudes and Aspirations on Educational Attainment and Participation. ResearchGate [Internet]. 2012. Available from: https://www.researchgate.net/publication/265288552_The_Impact_of_Attitudes_and_Aspirations_on_Educational_Attainment_and_Participation
63. Shalley CE, Oldham GR, Porac JF. Effects of Goal Difficulty, Goal-Setting Method, and Expected External Evaluation on Intrinsic Motivation. Academy of Management Journal [Internet]. 1987;30(3):533-563. Available from: https://www.proquest.com/docview/199822430?parentSessionId=sthWT7S5HHKtzY1zTogeVQ1CFMvskl2JSAHgK5sAiOs%3D&pq-origsite=primo&accountid=8424&sourcetype=Scholarly%20Journals
64. Garner B, Lally P, Wardle J. Making health habitual: the psychology of 'habit-formation' and general practice. British Journal of General Practice [Internet]. 2012;62(605):664-666. Available from: https://bjgp.org/content/62/605/664
65. Ogden J, Karim L, Choudry A, Brown K. Understanding successful behaviour change: the role of intentions, attitudes to the target and motivations and the example of diet. Health Education Research [Internet]. 2007;22(3):397-405. Available from: https://academic.oup.com/her/article/22/3/397/596170
66. Webb TL, Sheeran P. Does Changing Behavioral Intentions Engender Behavior Change? A Meta-Analysis of the Experimental Evidence. Psychological Bulletin [Internet]. 2006;132(2):249-268. Available from: https://oce-ovid-com.ezproxy.auckland.ac.nz/article/00006823-200603000-00004/HTML
